# Influence of Gelatin on Adhesion, Proliferation, and Adipogenic Differentiation of Adipose Tissue-Derived Stem Cells Cultured on Soy Protein–Agarose Scaffolds

**DOI:** 10.3390/foods13142247

**Published:** 2024-07-17

**Authors:** Seong-Joon Hong, Do-Hyun Kim, Ji-Hwan Ryoo, Su-Min Park, Hyuk-Cheol Kwon, Dong-Hyun Keum, Dong-Min Shin, Sung-Gu Han

**Affiliations:** 1 Department of Food Science and Biotechnology of Animal Resources, Konkuk University, Seoul 05029, Republic of Korea; rlfkrofkd@konkuk.ac.kr (S.-J.H.); secret311@konkuk.ac.kr (D.-H.K.); yjh9894@konkuk.ac.kr (J.-H.R.); psm207@konkuk.ac.kr (S.-M.P.); kwonhc@konkuk.ac.kr (H.-C.K.); illus4862@konkuk.ac.kr (D.-H.K.); 2 Department of Food Science and Technology, Keimyung University, Daegu 42601, Republic of Korea; sdm@kmu.ac.kr

**Keywords:** scaffold, gelatin, stem cell, adhesion, proliferation, adipogenic differentiation

## Abstract

Scaffolds play a key role in cultured meat production by providing an optimal environment for efficient cell attachment, growth, and development. This study investigated the effects of gelatin coating on the adhesion, proliferation, and adipogenic differentiation of adipose tissue-derived stem cells (ADSCs) cultured on soy protein–agarose scaffolds. Gelatin-coated scaffolds were prepared using 0.5% and 1.0% (*w*/*v*) gelatin solutions. The microstructure, water absorption rate, mechanical strength, cytotoxicity, cell adhesion, proliferation, and differentiation capabilities of the scaffolds were analyzed. Field emission scanning electron microscopy revealed the porous microstructure of the scaffolds, which was suitable for cell growth. Gelatin-coated scaffolds exhibited a significantly higher water absorption rate than that of non-coated scaffolds, indicating increased hydrophilicity. In addition, gelatin coating increased the mechanical strength of the scaffolds. Gelatin coating did not show cytotoxicity but significantly enhanced cell adhesion and proliferation. The gene expression levels of peroxisome proliferator-activated receptor gamma, CCAT/enhancer-binding protein alpha, and fatty acid-binding protein 4 were upregulated, and lipid accumulation was increased by gelatin coating. These findings suggest that gelatin-coated scaffolds provide a supportive microenvironment for ADSC growth and differentiation, highlighting their potential as a strategy for the improvement of cultured meat production and adipose tissue engineering.

## 1. Introduction

Meat is a major source of protein in the human diet. Global meat production and consumption are increasing because of improved living standards and population growth [1,2]. However, conventional meat production poses serious problems, including environmental pollution, animal welfare concerns, and antibiotic abuse [3]. Thus, sustainable alternatives for meat production are urgently needed. 

Cultured meat technology is emerging as an alternative to conventional meat production, with potential benefits such as reduced land use, lower greenhouse gas emissions, and lower energy consumption [3]. Cultured meat is produced by proliferating and differentiating muscle-derived stem cells (MSCs), adipose tissue-derived stem cells (ADSCs), and other types of cells [4]. Although numerous studies have used MSCs for cultured meat, ADSCs may also be useful in the development of cultured fat. The presence of fats in meat substantially influences the flavor, juiciness, and texture of the meat [5,6]. A lack of juiciness was shown in the sensory evaluation of cultured meat burgers which were only composed of skeletal muscle tissues [7]. 

A substantial number of cells and food-grade scaffolds are required to produce cultured fat. ADSCs are ideal for this purpose because of their accessibility, abundance, and ability to efficiently generate mature adipose tissues [8,9]. A scaffold is a three-dimensional structure made of biocompatible, biodegradable, and edible materials with suitable porosity and robustness, which supports early cell attachment and tissue formation [10,11,12,13,14]. Studies have shown that the structural integrity and porosity of scaffolds are crucial to facilitate nutrient and oxygen diffusion, which are essential for cell survival and proliferation [10,11,12,13,14]. Biocompatible and biodegradable materials, such as those used in our scaffolds, ensure that the scaffolds do not induce an adverse immune response and can be safely absorbed or excreted by the body over time. The robustness of the scaffold materials provides the necessary mechanical support to withstand the physical stresses during tissue formation, ensuring the scaffold maintains its structure and functionality throughout the cell growth and differentiation processes [10,11,12,13,14]. These characteristics are crucial to provide the necessary support and environment for cell growth. Hence, identifying appropriate materials that meet these requirements is necessary.

Soy proteins are highly biocompatible, similar to the extracellular matrix (ECM), and suitable for cell adhesion [15]. Previous studies have demonstrated that soy protein-based materials promote cell attachment, proliferation, and differentiation [16,17]. However, plant-derived proteins may lack sufficient structural integrity to maintain the desired scaffold shape and mechanical properties [18]. To overcome these limitations, proteins can be combined with polymers. This approach enhances their mechanical quality, water absorption, biocompatibility, and biological behavior, making them suitable for cultured food production.

Agarose is a natural polysaccharide and a common food additive that can enhance scaffold strength due to its stable framework at low concentrations [19]. Despite its outstanding biocompatibility and mechanical properties, agarose lacks cell-binding domains and does not support cell attachment [20]. Because of these limitations, crosslinking proteins and polysaccharides has been used to enhance stability and cell attachment [21]. In addition, cell adhesion to various scaffold surfaces is crucial to initiate signals that stimulate cell proliferation and differentiation [22].

Despite these advances in the development of scaffolds, the cell adhesion efficiency of stem cells remains a major concern. Gelatin derived from collagen is a promising solution because of its unique characteristics, including the presence of the RGD (Arg-Gly-Asp) tripeptide in its amino acid sequence. The RGD sequence, a cell recognition motif found in various ECM proteins, interacts with integrin receptors on the cell surface to promote cell attachment and mediate cell adhesion [23,24,25,26,27]. Integrin-mediated adhesion plays a crucial role in cellular communication and signaling, influencing key cellular behaviors such as migration, proliferation, and differentiation [23,24,25,26,27]. Studies have demonstrated that RGD sequences significantly enhance initial cell attachment to scaffolds, a critical step in tissue engineering [23,24,25,26,27]. This interaction not only anchors cells to the scaffold but also activates intracellular signaling pathways that promote cytoskeletal organization and gene expression associated with cell growth and differentiation. By incorporating RGD sequences, gelatin-coated scaffolds could mimic the natural ECM environment, thereby enhancing the efficiency of cell attachment and subsequent differentiation of cells [23,24,25,26,27]. Therefore, we hypothesized that gelatin coating of soy protein and agarose scaffolds can improve biocompatibility and cell attachment. To better understand these effects, our study aimed to investigate the impact of gelatin on the adhesion, proliferation, and differentiation of ADSCs on soy protein–agarose scaffolds for cultured fat production.

## 2. Materials and Methods

### 2.1. Materials

Soy protein isolate (SPI) was purchased from Shandong Yuxin Bio-Tech Co., Ltd. (Binzhou, China). Agarose was purchased from Fisher BioReagents (Pittsburgh, PA, USA). Gelatin powder was purchased from Sigma-Aldrich (St. Louis, MO, USA). Cell culture plates were purchased from SPL Life Sciences Co., Ltd. (Pocheon, Republic of Korea) and cell culture media were purchased from Gibco^TM^ (New York, NY, USA).

### 2.2. Preparation of Gelatin-Coated Soy Protein–Agarose Scaffolds

**Figure 1:**
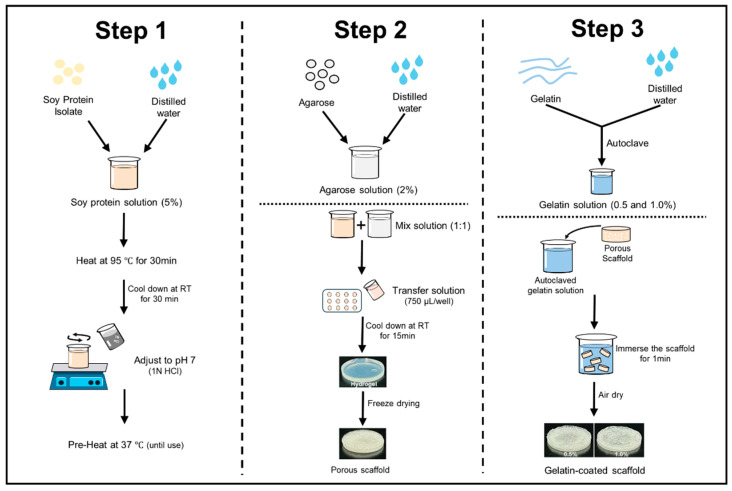
Fabrication steps of soy protein–agarose scaffolds and gelatin coating.

The scaffolds were prepared using SPI and agarose (Figure 1). The protein solution was prepared based on a previous report [28]. To prepare the protein solution, SPI was dissolved in distilled water at a concentration of 5% (*w*/*v*) and stirred at room temperature for 3 h. After stirring, the protein solution was hydrated at 4 °C for 24 h and then heated in a water bath at 95 °C for 30 min. The solution was cooled to 25 °C for 30 min with cold water, and the pH was adjusted to 7 using 1N HCl. The total volume was adjusted to 250 mL and kept at 37 °C until use. The agarose solution was prepared by dissolving agarose in distilled water at a concentration of 2% using a microwave [29]. Both solutions were then blended in a 1:1 ratio with a pre-warmed mixture of SPI. A hydrogel was then formed by transferring 750 µL of heated hydrogel solutions into each well of a 12-well plate and letting it cool for 15 min at room temperature. The hydrogels were stored overnight at −20 °C, then at −80 °C, and finally freeze-dried for 72 h. Subsequently, the scaffolds were immersed in a gelatin solution (0.5% and 1.0% *w*/*v*) for 1 min and allowed to solidify for 3 h at 4 °C. Finally, the scaffolds were air-dried for 2–3 h. Soy protein–agarose scaffolds without gelatin coating were used as the control. 

### 2.3. Field Emission Scanning Electron Microscopy (FE-SEM)

The porous morphology of the scaffolds was examined using FE-SEM (Hitachi SU8010, Hitachi Ltd., Tokyo, Japan). The scaffold samples were mounted on the FE-SEM support, sputtered with platinum using a sputter coater (Sputter Coater 180, Cressington Scientific Instruments, Watford, UK), and their morphologies were captured [30].

### 2.4. Water Absorption Rate

The water absorption rate was determined as reported previously [31]. The capacity of the scaffolds to absorb water was evaluated as follows: The scaffolds were first weighed to determine their dry weight (W_d_). Subsequently, the scaffolds were immersed in phosphate-buffered saline (PBS; Welgene Inc., Gyeongsan-si, Republic of Korea) at room temperature for 1, 3, 5, and 7 days. After each immersion period, the excess surface moisture was removed using Kimtech wipes (Yuhan Kimberly Ltd., Seoul, Republic of Korea), and the scaffolds were weighed again to determine their wet weight (W_w_). The water absorption (%) of each scaffold was calculated using the following formula:

(W_w_ − W_d_/W_d_) × 100 (%) (1)

### 2.5. Compressive Test

The mechanical characteristics of the scaffolds were investigated using a texture analyzer (TA-XT plus, Stable Micro Systems Ltd., Surrey, UK) in unconfined compression experiments. This experiment was conducted based on a previously reported study [11]. The scaffolds, 9 mm in diameter and 7 mm in height, were placed between two parallel stainless-steel plates. The stress response and elastic recovery were measured under 60% strain at a rate of 1.0 mm/s. Stress–strain curves were generated using the Exponent Connect software (Stable Micro Systems Ltd., Surrey, UK).

### 2.6. Isolation of Adipose Tissue-Derived Stem Cells (ADSCs)

ADSCs were isolated from the dorsal fat of 1-day-old Yorkshire piglets [32]. The adipose tissues were rinsed 2 to 3 times with PBS containing 10% antibiotic antifungal solution (AA; Gibco^TM^). The tissue was finely minced and digested with 0.2% collagenase type 2 (Sigma-Aldrich) in Dulbecco’s modified Eagle medium/Nutrient Mixture F-12 (DMEM/F-12), with continuous shaking at 100 rpm and 37 °C for 60 min. The resulting digestion medium was neutralized by adding Minimum essential medium alpha (MEM alpha) supplemented with 10% fetal bovine serum (FBS; Cytiva, Marlborough, MA, USA) and 1% A/A. The neutralized culture medium was filtered using a 100 μm cell strainer (Falcon, Corning Incorporated, Corning, NY, USA). The mixture was centrifuged at 250× *g* for 5 min at room temperature, and the cell pellet was carefully resuspended in MEM alpha supplanted with 10% FBS (Welgene Inc., Gyeongsan-si, Republic of Korea), 1% penicillin-streptomycin-glutamine (PSG; Gibco^TM^), and 10 ng/mL of basic fibroblast growth factor (bFGF; Bio-Techne, Minneapolis, MN, USA). After isolation, the ADSCs were cultured in a 100 mm culture plate.

### 2.7. Cell Culture Condition of ADSCs

In all tests, ADSCs from passages 3–6 were used and cultivated in a humidified incubator with 5% CO_2_ at 37 °C. FBS (10%), PSG (1% (*v*/*v*)), and bFGF (10 ng/mL) were added to MEMα as described in a previous article [32]. The cells were trypsinized and passaged once they reached approximately 80% confluence, and the growth medium was refreshed every 24 h. Adipogenic differentiation was conducted for 1, 7, and 14 days in low-glucose DMEM supplemented with 10% FBS, 1% PSG, 1 µM dexamethasone, 500 µM, 3-isobutyl-1-methylxanthine (IBMX), 10 µg/mL bovine insulin, and 100 µM indomethacin. The differentiation medium was replaced with fresh medium every 72 h.

### 2.8. Indirect Cytotoxicity Assay

Cytotoxicity assays were conducted with slight modifications based on a previous study [30,33]. The scaffolds were sterilized by overnight immersion in 70% ethanol, followed by 2 h of ultraviolet light exposure (1 h per side) and three washes with sterile PBS. The scaffolds were incubated at 37 °C with 5% CO_2_ for 1 day or 3 days in a scaffold–medium ratio of 1:4 (*v*/*v*) to prepare scaffold extracts. Prior to the assays, the cells were seeded in a 96-well plate at approximately 80% confluency. The growth medium was then replaced with liquid extracts from the scaffolds (1 day or 3 days) or a growth medium. After incubation for 24 h, the cytotoxicity of the scaffolds was analyzed using 3-(4,5-dimethylthiazol-2yl)-2,5-diphenyl-2H-tetrazolium bromide (MTT) and a lactate dehydrogenase (LDH) activity assay. 

#### 2.8.1. Cell Viability Test Using MTT Assay

Cell viability was evaluated using the MTT assay as described previously [33]. ADSCs were cultured in a 96-well plate and treated with liquid extracts from the scaffolds (1 day or 3 days) or growth for 24 h. After incubation, fresh medium containing 10 μL of MTT reagent (5 mg/mL in PBS) was added to each well and incubated for an additional 3 h. The medium was then removed, and acidic isopropanol was added to each well to dissolve the formazan crystals formed. The optical density (OD value) was measured at 570 and 630 nm using an Epoch microplate spectrophotometer (BioTek Instruments; Winooski, VT, USA). Cell viability was calculated by subtracting the OD at 630 nm from the OD at 570 nm, using the following formula:

(OD_Sample_/OD_control_) × 100 (%) (2)

#### 2.8.2. Cytotoxicity Test Using LDH Assay

To evaluate cellular damage, an LDH assay was performed using a CytoTox 96^®^ Non-Radioactive Cytotoxicity Assay kit (Promega; Madison, WI, USA) [30]. Cells were seeded in a 96-well plate and treated with the scaffold extracts (1 day or 3 days) or growth medium for 24 h. After treatment, the supernatants (50 μL/well) were transferred to a new 96-well plate, and CytoTox 96^®^ reagent (50 μL/well) was added. The plates were incubated for 30 min under light-free conditions. The OD was measured at 490 nm with a spectrophotometer. The LDH release rate was calculated using the following formula:

(OD_sample_ LDH release/OD_maximum_ LDH release) × 100. (3)

### 2.9. Seeding ADSCs on the Scaffolds, Assessment of Seeding Efficiency and Cell Proliferation

Prior to cell seeding, the scaffolds were sterilized as previously described and immersed in the medium for 1 h. After slightly removing the excess medium, 1 × 10^7^ cells were seeded per scaffold and allowed to adhere for 24 h, as described in a previous report [34]. The scaffolds were then filled with the growth medium, which was replaced daily. 

To evaluate the seeding efficiency, the number of ADSCs detached from the scaffolds was counted as described previously [9]. After 24 h of cell attachment, the scaffolds and medium were removed. The cells attached to the bottom of the wells were detached using trypsin-EDTA and counted using an optical microscope and a hemocytometer (Hausser Scientific, Horsham, PA, USA).

To assess the effect of gelatin coating on cell attachment and proliferation, DAPI staining was performed, as described previously [35]. After culturing the cells on the scaffolds for 1 day or 7 days, the culture medium was removed, and the cells were washed twice with PBS. The cells in the scaffolds were fixed in 4% paraformaldehyde, and the scaffolds were cut into 10 μm thick pieces and placed on slides. The scaffolds on the slides were then stained with DAPI (1 μg/mL) for 10 min and washed thrice with PBS. The cell nuclei were visualized, and the images were captured using a fluorescence microscope (Eclipse Ti2-U, Nikon Co., Ltd., Tokyo, Japan) and Nikon Eclipse Ts2R camera (Nikon Co., Ltd., Tokyo, Japan).

### 2.10. Oil Red O Staining

Oil Red O staining was used to visually analyze the adipogenic differentiation of ADSCs in the scaffolds following differentiation induction. Oil Red O staining with scaffolds was performed following the methods described in a previous study [9]. The scaffold samples were fixed using 4% paraformaldehyde for 1 h. Subsequently, the fixed samples were embedded in the optimal cutting temperature tissue freezing mixture and cut into 10 μm thick slices. The slices were placed on the slides and stained with an Oil Red O working solution for 10 min at room temperature. An Oil Red O stock solution (3.5 g/L) was prepared in isopropanol and diluted 3:2 (*v*:*v*) with deionized water. The solution was incubated at room temperature for 20 min and then filtered through a 0.22 μm filter. After being rinsed using deionized water four times, samples were visualized using Nikon Eclipse Ti2-U microscope and a Nikon Eclipse Ts2R camera.

### 2.11. Determination of the mRNA Levels of Adipogenic Differentiation Markers by RT-PCR

The mRNA expression of genes associated with adipogenic differentiation of ADSCs, including peroxisome proliferator-activated receptor gamma (*PPARγ*), CCAT/enhancer-binding protein alpha (*C*/*EBPα*), and fatty acid-binding protein 4 (*FABP4*), in the scaffolds was measured by RT-PCR. To determine gene expression, RNA samples from the scaffolds were extracted using TRIzol reagent (Ambion, Austin, TX, USA). The TOPscript RT DryMIX kit (Enzynomics, Daejeon, Korea) was used to synthesize cDNA. RNA extraction, cDNA synthesis, and RT-PCR were performed following the methods described in a previous study [36]. The mRNA levels were evaluated using the Roche LightCycler 96^®^ System (Basel, Switzerland) and 2× Real-Time PCR mix (BIOFACT CO., Ltd., Daejeon, Republic of Korea). Thermal cycling was performed at 95 °C for 15 min, followed by 60 cycles of denaturation (10 s at 95 °C), annealing (10 s at 60 °C), and extension (10 s at 72 °C). The mRNA level was corrected using the ΔΔCq method and glyceraldehyde 3-phosphate dehydrogenase (*GAPDH*) mRNA level. The primers used for RT-PCR are shown in Table 1.

### 2.12. Statistical Analysis

All experiments were carried out in triplicate, and the results were expressed as the mean ± standard error using the SPSS-PASW Ver. 22.0 (SPSS Inc., Chicago, IL, USA). Statistical significance was evaluated using one-way ANOVA and independent two-sample *t*-tests, with *p* < 0.05 considered significant.

## 3. Results

### 3.1. Water Absorption Rate of the Scaffolds

**Figure 2:**
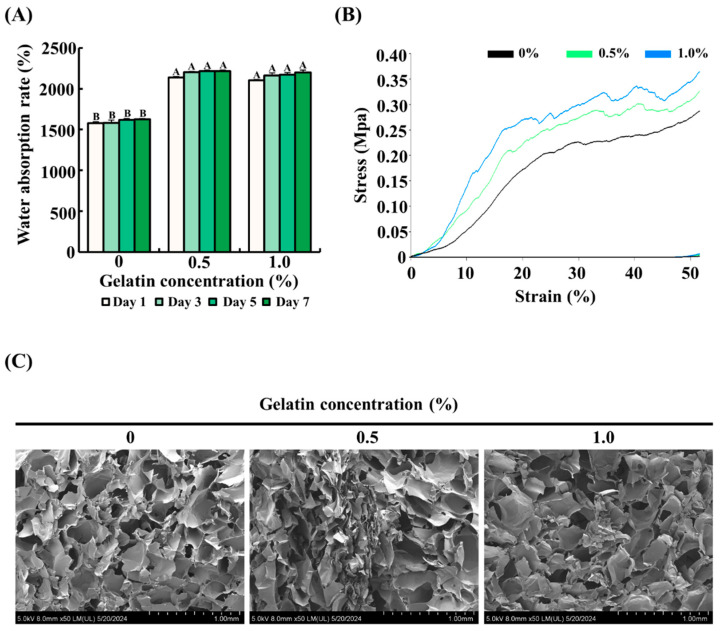
Effect of gelatin coating on the physical properties of soy protein–agarose scaffolds. The scaffold without gelatin coating was used as control. (**A**) Water absorption rate of the scaffolds. (**B**) Mechanical strength of the scaffolds. (**C**) Porous structure of the scaffolds. Cross section of the scaffolds was captured using field emission electron microscopy. All experiments were performed at least three times. (**A**,**B**) Different letters indicated a significant difference in water absorption rates as determined by Tukey’s post hoc test (*p* < 0.05). The data are expressed as the mean ± standard error of the mean (*n* = 6).

To determine the effect of gelatin on the water absorption rate of the scaffolds, the scaffolds with gelatin coating (0.5% or 1.0%) were compared with uncoated scaffolds. As shown in Figure 2A, uncoated scaffolds absorbed approximately 1500% water compared with their dry weight. In comparison, the gelatin-coated scaffolds showed a significantly higher water absorption rate, approximately 1.5 times greater than the control group, reaching around 2250% of their dry weight (*p* < 0.05).

### 3.2. Mechanical Strength of the Scaffolds

The effect of gelatin coating on the mechanical properties of soy protein–agarose scaffolds was assessed using a compressive test. As depicted in Figure 2B, the gelatin-coated scaffolds exhibited improved mechanical strength compared with the control group. Specifically, the mechanical strength increased proportionally with the concentration of gelatin, as evidenced by the higher stress values at various strain levels. These results suggest that gelatin coating formed a composite structure around the scaffold and enhanced its mechanical strength.

### 3.3. Microstructure of the Scaffolds

The impact of gelatin coating on the microstructure of soy protein–agarose scaffolds was examined using FE-SEM. As shown in Figure 2C, both gelatin-coated and control groups had a porous microstructure with interconnected pores, indicating that gelatin coating did not affect the internal porous structure.

### 3.4. Cytotoxicity Evaluation of Scaffolds

**Figure 3:**
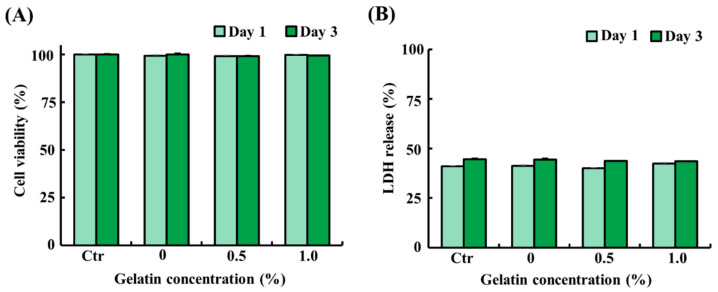
Cytotoxicity of the soy protein–agarose scaffolds and gelatin-coated soy protein–agarose scaffolds. Adipose tissue-derived stem cells (ADSCs) from Yorkshire piglets were treated with the liquid extracts from the scaffolds. The liquid extracts were prepared by incubating the scaffolds in a medium at a scaffold-to-medium ratio of 1:4 for 24 h. The cells were treated with the liquid extracts from the scaffolds for 1 day and 3 days. (**A**) Cell viability of ADSCs treated with the liquid extracts from the scaffolds. (**B**) Lactate dehydrogenase (LDH) release from ADSCs treated with the liquid extract from the scaffolds. All experiments were repeated at least three times. The data are expressed as the mean ± standard error of the mean (*n* = 3).

To assess the potential cytotoxicity of the scaffolds, ADSCs were exposed to liquid extracts from the scaffolds (control and 0.5% and 1.0% gelatin-coated) for 1 day and 3 days. As shown in Figure 3A,B, ADSCs treated with the liquid extracts from the scaffolds showed similar cell viability and LDH release as that of the control (*p* > 0.05). The results demonstrated that both gelatin-coated and uncoated scaffolds exhibit excellent biocompatibility, as evidenced by 100% cell viability. These findings suggest that the scaffolds do not adversely affect cell survival or proliferation, thereby establishing their suitability for applications in cultured fat production.

### 3.5. Seeding Efficiency and Proliferation of ADSCs on the Scaffolds

**Figure 4:**
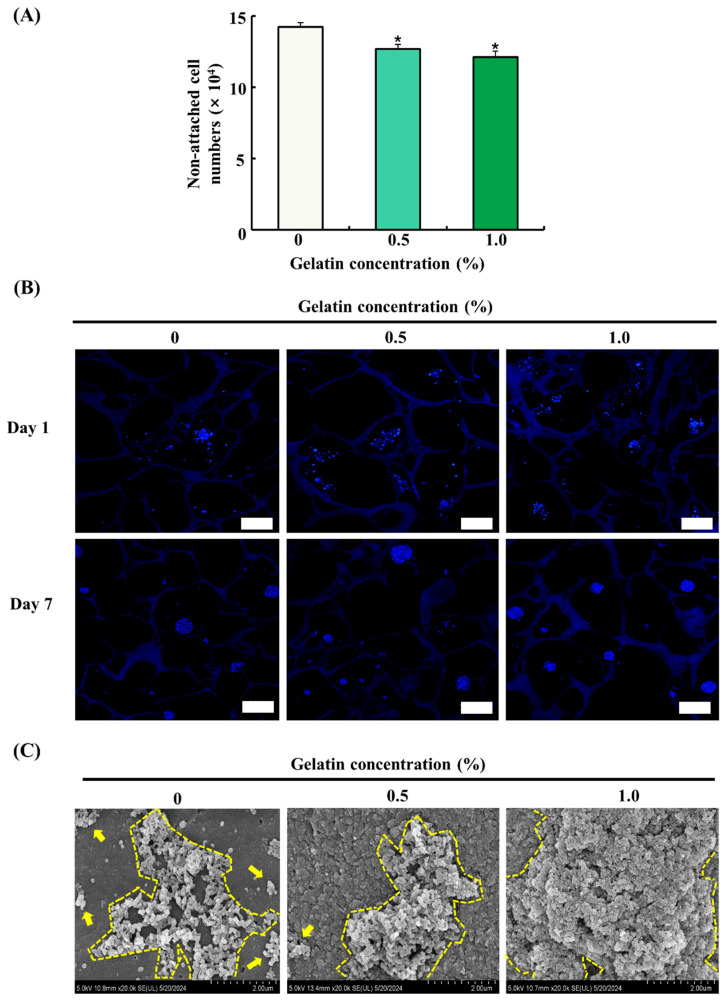
Effects of gelatin coating on soy protein–agarose scaffolds regarding the adhesion and proliferation of adipose tissue-derived stem cells (ADSCs). The scaffold without gelatin was used as the control. (**A**) The effect of gelatin coating on initial cell seeding efficiency was evaluated by measuring the number of non-attached cells (×10^4^) at different gelatin concentrations (0%, 0.5%, and 1.0%) (*n* = 4). (**B**) The effects of gelatin coating on ADSC adhesion and proliferation were evaluated at different gelatin concentrations (0%, 0.5%, and 1.0%) and time points (day 1 and 7) with DAPI staining. The scaffolds were prepared by cutting into 10 μm thick sections. The magnification of images is 100×. The scale bar indicates 100 µm. (**C**) The effects of gelatin coating on adhesion and proliferation of ADSCs were evaluated at different gelatin concentrations (0%, 0.5%, and 1.0%) on day 7 using field emission electron microscopy. The scalebar indicates 2.0 µm. Representative images were selected from three independent replicates. Data are expressed as the mean ± standard error of the mean. * (*p* < 0.05) shows a significant difference compared to the control group.

The attachment and proliferation of ADSCs on the scaffolds were evaluated by cell counting using a hemocytometer, DAPI staining, and FE-SEM imaging. The seeding efficiency of ADSCs was determined by counting the number of cells in cultured medium 24 h after seeding. As shown in Figure 4A, the gelatin-coated groups exhibited a significant decrease in the number of non-attached cells compared with that of the control group (*p* < 0.05), indicating enhanced cell adhesion. However, no significant difference was observed between the gelatin-coated groups. DAPI staining of ADSCs on the scaffolds on day 1 and 7 showed that the adhesion and proliferation of ADSCs were higher in the gelatin-coated groups than those in the control group, as shown in Figure 4B. The FE-SEM images showed no significant increase in cell proliferation in the 0.5% gelatin-coated group, but a significant increase was observed in the 1.0% gelatin-coated group compared with the control group (Figure 4C).

### 3.6. Effect of Gelatin Coating on the Expression of Adipogenic Differentiation-Related Genes in Soy Protein–Agarose Scaffolds

**Figure 5:**
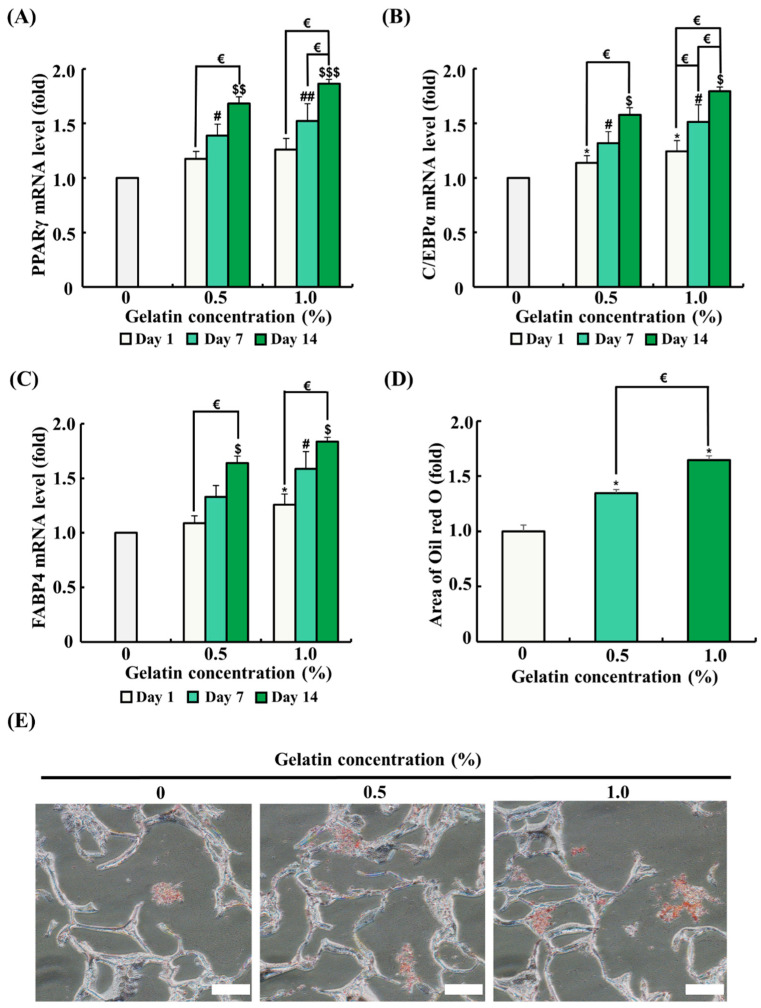
Effects of gelatin coating on adipogenic differentiation of adipose tissue-derived stem cells (ADSCs) in soy protein–agarose scaffolds. The scaffold without gelatin coating was used as control. (**A**–**C**) The effects of gelatin coating on ADSC adipogenic differentiation were evaluated at different gelatin concentrations (0%, 0.5%, and 1.0%) using real-time polymerase chain reaction (RT-PCR) at different time points (day 1, 7, and 14). The expression levels of the adipogenic differentiation markers peroxisome proliferator-activated receptor gamma (*PPARγ*), CCAAT/enhancer-binding protein alpha (*C*/*EBPα*), and fatty acid-binding protein 4 (*FABP4*) were assessed (*n* = 3). (**D**,**E**) The effects of gelatin coating on lipid accumulation in soy protein–agarose scaffolds were evaluated using Oil Red O staining after the adipogenic differentiation of ADSCs. The scaffolds were prepared by cutting 10 μm thick sections. The evaluation was conducted at various gelatin concentrations (0%, 0.5%, and 1.0%) following a 14-day differentiation period. Representative images were selected from three independent replicates. The magnification of images is 200×. Scale bar indicates 100 µm. Data are expressed as the mean ± standard error of the mean. * (*p* < 0.05) shows significant differences between the control group and gelatin-coated groups on day 1. # (*p* < 0.05) and ## (*p* < 0.01) show significant differences between the control group and gelatin-coated groups on day 7. $ (*p* < 0.05), $$ (*p* < 0.01), and $$$ (*p* < 0.001) show significant differences between the control group and gelatin-coated groups on day 14. € (*p* < 0.05) shows a significant difference between the gelatin-coated groups.

The mRNA expression of genes related to adipogenic differentiation was analyzed by quantitative RT-PCR. ADSCs were seeded on the scaffolds and differentiated for 1, 7, and 14 days. As the differentiation period progressed, mRNA expression of genes associated with adipogenic differentiation (*PPARγ*, *C/EBPα*, and *FABP4*) significantly increased (Figure 5A–C). The 0.5% gelatin coating significantly increased the mRNA expression of *C/EBPα* on day 1 (*p* < 0.05) and significantly increased the mRNA expression of *PPARγ*, *C/EBPα*, and *FABP4* on day 7 and day 14 over time (*p* < 0.05) (Figure 5A–C). Meanwhile, the 1.0% gelatin coating significantly increased the mRNA expression of *C/EBPα* and *FABP4* in ADSCs on day 1 (*p* < 0.05) (Figure 5A–C). Similar to the results of the 0.5% gelatin coating, the 1.0% gelatin coating significantly increased the mRNA expression of *PPARγ*, *C/EBPα*, and *FABP4* in ADSCs on day 7 and day 14 (*p* < 0.05) (Figure 5A–C).

### 3.7. Effect of Gelatin Coating on Lipid Accumulation in Soy Protein–Agarose Scaffolds

Oil Red O staining was conducted to evaluate the effect of gelatin coating on adipogenic differentiation after 14 days in an adipogenic differentiation medium. In Oil Red O staining, an increased red-stained area correlates with higher lipid accumulation and indicates enhanced adipogenic differentiation. As shown in Figure 5E, ADSCs in the gelatin-coated groups showed a significant increase in the red-stained area compared with that in the control, suggesting that gelatin coating promotes adipogenic differentiation. 

## 4. Discussion

Cultured meat technology uses three-dimensional scaffolds to support cell growth and tissue formation [14]. Plant-based proteins, such as soy protein, provide biocompatibility and cost-effectiveness to the scaffolds, resembling the ECM that supports cell adhesion and growth. However, to improve the mechanical strength and stability of soy protein scaffolds, they are often blended with polymers, such as agarose, a polysaccharide that effectively improves the integrity of the scaffolds [15]. 

Current research has primarily focused on muscle tissue-derived cells for cultured meat production. However, fat is crucial for flavor and texture, and research on cultured fat is essential for mimicking conventional meat [5,7,37]. Therefore, we used ADSCs for adipogenic differentiation [8,9]. The efficiency of the initial cell attachment is important for increasing the production yield of cultured muscle or fat. Although plant-based materials were mainly used in our scaffolds, modifications are necessary to enhance cell attachment on the scaffolds [29]. In fact, the ECM can improve cell attachment, as it contains cell adhesion sites known as RGD motifs. However, due to the high cost of the ECM, gelatin was selected as a coating material. Gelatin is more affordable and structurally similar to the ECM and therefore enhances cell attachment.

Previous studies have emphasized the importance of edible scaffolds with adequate structural stability, high water-holding capacity, and porous structures for cultured meat [10,11,12]. Therefore, we evaluated the physical properties of the scaffolds using the water absorption rate, compressive test, and FE-SEM imaging. Our results revealed that the gelatin-coated groups exhibited a significantly higher water absorption capacity than that of the control, without affecting the porosity. Similarly, other studies have reported that gelatin coating improves the water absorption of scaffolds [38]. In this study, a higher concentration of gelatin was used, which may have resulted in higher water uptake due to the greater number of hydrophilic groups, such as OH and NH_2_ [39,40]. Additionally, our study found that the mechanical strength of the scaffolds increased in a concentration-dependent manner with gelatin coating. Other research has shown similar data that mechanical strength improves with higher gelatin concentrations [38]. This may be due to the fact that the enveloping gelatin can create a stronger composite structure. This robust composite structure may help in forming a more stable environment, which is beneficial for cultured fat production [41]. These enhanced physical characteristics also suggest that gelatin-coated scaffolds may provide a more suitable microenvironment for cell growth and differentiation in the context of cultured fat tissue engineering.

The cytotoxicity assessment of the scaffolds followed the ISO 10993-5 guidelines, which are widely used for the evaluation of material biocompatibility [30,33,42]. ADSCs were exposed to the liquid extracts from the scaffolds for 1 day and 3 days, and cell viability and LDH release were measured. The results showed no significant differences in these parameters in ADSCs between the scaffold extracts and control group on either day. Like our findings, a previous study demonstrated that cell viability rates approached 100% in various treatments [43]. These results indicate that soy protein–agarose scaffolds and gelatin are non-toxic and biocompatible materials. Therefore, these materials have the potential to be used as scaffolds for cultured fat production.

Cell adhesion and proliferation were assessed through non-attached cell counting, DAPI staining, and FE-SEM analysis. The gelatin-coated groups exhibited a significant reduction in non-attached cells compared with the control, indicating enhanced cell adhesion. Moreover, DAPI staining and FE-SEM revealed a significant increase in cell attachment and proliferation on the gelatin-coated scaffolds. This enhancement in cell adhesion and proliferation can be attributed to the presence of the RGD (Arg-Gly-Asp) tripeptide sequence inherent in gelatin. RGD is a well-known cell recognition motif found in various ECM proteins, facilitating cell adhesion by interacting with integrin receptors on the cell surface [24,25,26]. Integrins are transmembrane receptors that mediate the attachment between the cells and their surroundings, including other cells and the ECM [27]. The RGD–integrin interactions play a crucial role in mediating cell adhesion to biomaterials and promoting cell proliferation and differentiation [24]. In our study, increased cell proliferation in the gelatin-coated scaffolds was observed, and this may be due to the presence of bioactive RGD motifs within the gelatin. Our findings suggest that gelatin coating could be an effective strategy for the increase in cultured fat production by supporting cell adhesion and proliferation.

We also investigated the effect of gelatin coating on soy protein–agarose scaffolds regarding adipogenic differentiation of ADSCs. Gelatin coating significantly enhanced adipogenesis by upregulating key adipogenic markers, *PPARγ*, *C*/*EBPα*, and *FABP4*. The mRNA expression levels of these markers progressively increased over time, indicating sustained support for adipogenic differentiation by gelatin coating. During the initial period, gelatin coating significantly increased the mRNA expression of specific adipogenic differentiation markers, with the 0.5% gelatin coating increasing *C*/*EBPα* and the 1.0% gelatin coating increasing both *C*/*EBPα* and *FABP4*. As the differentiation period extended to day 7 and 14, the mRNA expression of adipogenic markers (*PPARγ*, *C*/*EBPα*, and *FABP4*) was significantly upregulated. Adipogenic differentiation was induced for 14 days, and lipid accumulation in ADSCs was observed using Oil Red O staining. Oil Red O staining data showed that gelatin coating significantly increased lipid accumulation compared with the control group. This increase in adipogenic differentiation may be due to the increase in cell attachment, cell proliferation, and the RGD motif of gelatin. Previous studies have demonstrated that RGD-modified hydrogels exhibit enhanced adipogenic activity [44]. Similarly, we found that gelatin coating increased the mRNA expression of differentiation-related genes and lipid accumulation in cells, suggesting that the RGD sequences contained in gelatin mediate adipogenic activity. These results suggest that gelatin coating exerts a positive effect on adipogenic differentiation and can be used as a method to enhance the production of cultured fat.

## 5. Conclusions

Our study demonstrated that gelatin coating on soy protein–agarose scaffolds significantly enhanced both physical and biological properties, leading to improved cell attachment, proliferation, and differentiation of ADSCs. The gelatin coating enhanced the water absorption capacity and mechanical strength of the scaffolds while maintaining biocompatibility. The enhanced cell adhesion and proliferation of ADSC on gelatin-coated scaffolds were attributed to the presence of RGD motifs in gelatin. Upregulation of adipogenic markers, including *PPARγ*, *C*/*EBPα*, and *FABP4*, demonstrated by increased lipid accumulation, indicates effective adipogenic differentiation. Overall, this approach offers a viable strategy for the production of cultured adipose tissue, contributing to more sustainable meat alternatives and advancing its applications in cultured food products. However, the mechanism by which gelatin promotes adipogenic differentiation remains unclear, and further research is required to understand the precise mechanism.

## Figures and Tables

**Table 1:** Primers used for RT-PCR.

Gene	Primer Sequence (5′→3′)	
*PPARγ*	F	AGGTGGCCATTCGCATCTTTCA
	R	TCGTGGACGCCATACTTTAGGAGA
*C*/*EBPα*	F	GGGGCTTTGAACCTAAGGTTGT
	R	GAACATATGGCCTCAGAGCTAACC
*FABP4*	F	TGGCCAAACCCAACCTGATCAT
	R	GGTGCTCTTGACTTTCCTGTCATC
*GAPDH*	F	ATGACCCCTTCATTGACCTCCACT
	R	ACCAGCATCGCCCCATTTGATT

*PPARγ*, peroxisome proliferator-activated receptor gamma; *C*/*EBPα*, CCAAT/enhancer-binding protein-alpha; *FABP4*, fatty acid-binding protein 4; *GAPDH*, glyceraldehyde 3-phosphate dehydrogenase.

## Data Availability

The original contributions presented in the study are included in the article, further inquiries can be directed to the corresponding author.
